# Supplementary material for: Temporal Distribution Patterns of Cryptic Brachionus calyciflorus (Rotifera) Species in Relation to Biogeographical Gradient Associated with Latitude
Source: Animals (Basel). 2024 Jan 12;14(2):244. doi: 10.3390/ani14020244 (PMC10812649; doi:10.3390/ani14020244)
Supplement: Supplementary file 1 [file animals-14-00244-s001.zip › Supplementary Table S1.pdf]

Table S1. Shared mtCOI haplotypes among all 790 individuals of *B. calyciflorus* complex.

Sampling location	Shared hapltype	Individuals
Lake	YCSH1	YL01_14, YL01_34, YL02_80, YL03_5
Yunlong	YCSH2	YL01_17, YL01_23, YL02_81, YL04_8
	YCSH3	YL02_5, YL03_21
	YCSH4	YL04_16, YL04_19
	YCSH5	YL06_1, YL06_15, YL06_16, YL06_21, YL06_23, YL06_25, YL06_27, YL06_28, YL06_3, YL06_30, YL06_32, YL06_33, YL06_35, YL06_36, YL06_38, YL06_4, YL06_41, YL06_42, YL06_44, YL06_7, YL06_8, YL07_10, YL07_14, YL07_19, YL07_2, YL07_26, YL07_27, YL07_28, YL07_29, YL07_30, YL07_35, YL07_44, YL07_45, YL07_47, YL07_7, YL07_8, YL08_10, YL08_19, YL08_22, YL08_26, YL08_28, YL08_30, YL08_31, YL08_34, YL08_37, YL08_39, YL08_8, YL08_9, YL09_1, YL09_10, YL09_11, YL09_12, YL09_13, YL09_16, YL09_17, YL09_18, YL09_19, YL09_2, YL09_20, YL09_3, YL09_4, YL09_5, YL09_6
	YCSH6	YL06_18, YL06_2, YL07_23, YL07_3, YL07_34, YL07_6, YL08_12, YL09_8, YL09_9
	YCSH7	YL06_24, YL06_39, YL06_40, YL07_39, YL09_15
	YCSH8	YL06_34, YL08_23, YL08_33
	YCSH9	YL11_6, YL12_15, YL12_16, YL02_55
	YCSH10	YL12_1, YL12_13, YL01_27
	YCSH11	YL12_10, YL01_10, YL01_13
	YCSH12	YL12_21, YL01_15, YL04_2
	YCSH13	YL12_22, YL12_23
	YCSH14	YL12_44, YL03_19
	YCSH15	YL12_9, YL01_11, YL01_25, YL02_65
Lake	JCSH1	JH01_11, JH01_19, JH01_7, JH02_12, JH03_3, JH03_36
Jinghu	JCSH2	JH01_5, JH02_37
	JCSH3	JH04_17, JH04_24, JH05_7, JH07_5
	JCSH4	JH04_20, JH05_11, JH09_1, JH09_34
	JCSH5	JH04_34, JH04_7, JH05_3
	JCSH6	JH04_46, JH05_12, JH05_20, JH07_20, JH07_46
	JCSH7	JH04_9, JH06_10, JH06_15, JH06_22, JH06_35, JH08_1, JH08_10, JH08_14, JH08_15, JH08_18, JH08_19, JH08_22, JH08_3, JH08_4, JH08_5, JH08_7
	JCSH8	JH06_12, JH06_38, JH06_8
	JCSH9	JH06_13, JH06_14, JH06_18, JH06_2, JH06_20, JH06_24, JH06_26, JH06_27, JH06_28, JH06_29, JH06_3, JH06_34, JH06_37, JH06_40, JH06_41, JH06_42, JH06_45, JH06_6, JH07_11, JH08_12, JH08_17, JH08_21, JH08_23, JH09_11, JH09_16, JH09_3, JH09_7
	JCSH10	JH10_11, JH10_36, JH04_10, JH04_15, JH04_21, JH04_22, JH04_28, JH04_29, JH04_43, JH04_47, JH05_16, JH05_21
	JCSH11	JH10_22, JH04_30, JH04_45, JH06_1, JH07_36, JH09_29
	JCSH12	JH11_22, JH06_11, JH06_17, JH06_23, JH06_25, JH06_30, JH06_44, JH06_47, JH06_48, JH08_6, JH09_23
Lake	HCSH1	JN03_14, JN03_21, JN03_27, JN03_36, JN03_41, JN03_47, JN03_48
Jinniu	HCSH2	JN05_27, JN05_44, JN05_7, JN07_17, JN07_30, JN07_43, JN08_11, JN08_15, JN08_17, JN08_20, JN08_29, JN09_12, JN09_13, JN09_16, JN09_20, JN09_21,

Sampling location	Shared hapltype	Individuals
		JN09_22, JN09_25, JN09_34, JN09_45, JN11_17, JN11_9, JN01_10, JN01_15, JN01_17, JN01_18, JN01_9
	HCSH3	JN05_32, JN05_48, JN11_45, JN12_13, JN12_4, JN12_9, JN01_13, JN01_16, JN01_2, JN01_4, JN01_5, JN01_7, JN02_42
	HCSH4	JN12_11, JN12_12, JN12_14, JN12_15, JN12_17, JN12_18, JN12_19, JN12_22, JN12_3, JN12_5, JN12_7, JN12_8, JN01_14, JN01_3, JN01_6
Lake Jinghu & Lake Jinniu	JHCSH1	JH06_16, JH06_19, JH06_4, JH06_7, JH07_10, JH07_32, JH07_9, JH08_25, JH09_13, JH09_18, JN12_20, JN05_1, JN05_28, JN05_40, JN05_45
	JHCSH2	JH10_16, JH10_17, JN10_31, JN10_38, JN10_42, JN10_44, JN11_14, JN11_7, JN02_1, JN02_12, JN02_17, JN02_18, JN02_22, JN02_23, JN02_25, JN02_26, JN02_30, JN02_32, JN02_33, JN02_35, JN02_41, JN02_43, JN02_46, JN02_47, JN02_48, JN02_6, JN03_31, JN09_18, JN09_24, JN09_30, JN09_32, JN09_33, JN09_35, JN09_41, JN09_44
	JHCSH3	JH10_18, JN10_28, JN10_32, JN10_37, JN11_33, JN11_31, JN11_25, JN11_24, JN12_16, JN12_6, JN02_40, JN03_10, JN03_11, JN03_12, JN03_13, JN03_16, JN03_18, JN03_19, JN03_22, JN03_23, JN03_24, JN03_25, JN03_29, JN03_3, JN03_32, JN03_33, JN03_34, JN03_35, JN03_37, JN03_38, JN03_39, JN03_4, JN03_40, JN03_43, JN03_44, JN03_6, JN03_7, JN03_8, JN03_9, JN04_1, JN04_11, JN04_12, JN04_13, JN04_19, JN04_2, JN04_20, JN04_22, JN04_25, JN04_26, JN04_3, JN04_48, JN04_6, JN04_9, JN05_15, JN05_26, JN06_10, JN06_11, JN06_13, JN06_14, JN06_16, JN06_17, JN06_19, JN06_20, JN06_21, JN06_3, JN06_6, JN06_8, JN07_27
Lake Jinghu & Lake Yunlong	JYCSH1	JH10_1, JH10_10, JH10_12, JH10_13, JH10_14, JH10_28, JH10_3, JH10_4, JH10_5, JH10_6, JH10_9, JH11_13, JH11_14, JH11_15, JH11_16, JH11_18, JH11_20, JH11_21, JH04_1, JH04_11, JH04_12, JH04_14, JH04_16, JH04_18, JH04_2, JH04_23, JH04_26, JH04_27, JH04_33, JH04_35, JH04_36, JH04_37, JH04_38, JH04_39, JH04_4, JH04_40, JH04_41, JH04_42, JH04_5, JH04_6,JH05_13, JH05_15, JH05_17, JH05_18, JH05_2, JH05_5, JH05_6, JH05_8, JH06_32, JH06_36, JH06_9, JH07_12, JH07_13, JH07_19, JH07_2, JH07_21, JH07_24, JH07_27, JH07_29, JH07_3, JH07_30, JH07_31, JH07_35, JH07_38, JH07_4,JH07_43, JH07_44, JH07_45, JH07_6, JH07_8, JH09_10, JH09_14, JH09_21, JH09_25, JH09_31, JH09_4, JH09_8, YL06_43, YL09_14, YL09_21
	JYCSH2	JH11_19, JH11_17, JH11_25, JH11_26, JH11_30, JH11_31, JH11_32, YL12_34
	JYCSH3	JH11_23, JH02_39, YL12_33
	JYCSH4	JH12_12, YL12_20, YL12_26, YL12_3, YL12_36, YL12_8, YL01_33, YL02_50, YL02_85, YL03_13, YL03_22, YL03_7, YL04_14, YL04_22, YL04_27
	JYCSH5	JH12_15, JH12_17, JH12_23, JH12_24, JH12_29, JH12_36, JH12_6, JH01_13, JH01_16, JH01_18, JH01_20, JH01_25, JH01_4, JH01_8, JH02_11, JH02_22, JH02_26, JH02_36, JH02_9, JH03_13, JH03_15, JH03_17, JH03_18, JH03_19, JH03_22, JH03_39, YL11_4, YL11_5, YL12_11, YL12_2, YL12_24, YL12_31, YL12_38, YL12_48, YL01_31, YL02_12, YL02_60, YL03_18, YL04_28
	JYCSH6	JH12_18, JH12_19, JH12_21, JH12_26, JH12_30, JH12_34, JH12_35, JH01_1, JH01_10, JH01_12, JH01_17, JH01_21, JH01_22, JH01_23, JH02_20, JH02_23, JH02_3, JH02_30, JH03_11, JH03_20, JH03_28, JH03_29, JH03_30, JH03_31, JH03_32, JH03_8, YL12_18, YL12_19, YL12_25, YL12_32, YL12_40, YL12_41,

Sampling location	Shared hapltype	Individuals
		YL12_42, YL12_43, YL01_16, YL01_26, YL01_35, YL01_5, YL01_8, YL01_9, YL02_51, YL02_54, YL02_59, YL02_68, YL02_72, YL02_74, YL02_84, YL03_1, YL03_11, YL03_2, YL03_23, YL03_28, YL03_29, YL03_4, YL03_8, YL04_11, YL04_12, YL04_15, YL04_20, YL04_34, YL04_4, YL04_5, YL04_9
Lake Yunlong & Lake Jinniu	HYCSH1	JN01_1, YL10_49, YL10_50
	HYCSH2	JN05_14, JN05_17, JN05_21, JN05_24, JN05_33, JN05_47, JN05_9, JN07_16, JN07_25, JN07_42, JN07_44, JN08_1, JN08_13, JN08_19, JN08_31, JN08_6, JN09_14, JN09_29, JN09_37, JN09_43, JN09_9, YL05_12, YL05_14, YL05_25, YL05_28, YL05_30, YL05_33, YL05_38, YL05_46
	HYCSH3	JN08_10, JN08_9, JN09_11, JN09_15, JN09_23, JN09_38, JN09_39, YL10_40, YL10_9, YL05_1, YL05_13, YL05_15, YL05_18, YL05_19, YL05_20, YL05_21, YL05_24, YL05_29, YL05_32, YL05_4, YL05_42, YL05_43, YL05_5, YL05_6, YL05_8, YL06_49, YL06_6, YL07_22
Lake Yunlong, Lake Jinghu & Lake Jinniu	ACSH	JN05_11, JN05_12, JN05_13, JN05_16, JN05_19, JN05_2, JN05_20, JN05_22, JN05_23, JN05_25, JN05_29, JN05_30, JN05_34, JN05_35, JN05_36, JN05_39, JN05_42, JN05_46, JN05_6, JN05_8, JN07_10, JN08_21, JN08_8, JN09_31, YL10_11, YL10_12, YL10_15, YL10_17, YL10_18, YL10_22, YL10_25, YL10_29, YL10_31, YL10_35, YL10_51, YL10_52, YL10_53, YL10_54, YL10_55, YL10_56, YL10_57, YL10_6, YL10_8, YL11_1, YL11_10, YL11_12, YL05_10, YL05_23, YL05_26, YL05_34, YL06_29, YL07_40, YL08_1, YL08_11, YL08_13, YL08_14, YL08_2, YL08_20, YL08_21, YL08_25, YL08_27, YL08_29, YL08_3, YL08_32, YL08_35, YL08_36, YL08_38, YL08_4, YL08_41, YL08_42, YL08_43, YL08_44, YL08_45, YL08_47, YL08_48, YL08_5, YL08_6, JH04_13, JH04_3

CSH represents shared haplotype, and the other codes are abbreviations for geographic locations corresponding to Supplementary Table 1
